# Supplementary material for: Healthcare utilization during the first two waves of the COVID-19 epidemic in South Africa: A cross-sectional household survey
Source: PLoS One. 2023 Aug 25;18(8):e0290787. doi: 10.1371/journal.pone.0290787 (PMC10456221; doi:10.1371/journal.pone.0290787)
Supplement: Attachment — Submitted filename: Response to Reviewers.docx [file pone.0290787.s001.docx]

**Response to Reviewers**

Manuscript number: PONE-D-23-03137

Manuscript title: Healthcare utilization during the first two waves of the COVID-19 epidemic in South Africa: a cross-sectional household survey

Thank you for the feedback from the reviewers, which has been helpful in improving the manuscript. All references to page numbers are based on the track changes version of the revised manuscript.

**Journal Requirements:**

When submitting your revision, we need you to address these additional requirements.

1. Please ensure that your manuscript meets PLOS ONE's style requirements, including those for file naming. The PLOS ONE style templates can be found at

https://journals.plos.org/plosone/s/file?id=wjVg/PLOSOne_formatting_sample_main_body.pdf and

<https://journals.plos.org/plosone/s/file?id=ba62/PLOSOne_formatting_sample_title_authors_affiliations.pdf>

**Reply:** The revised manuscript has been edited to meet PLOS ONE's style requirements.

2. Thank you for stating the following in the Competing Interests/Financial Disclosure * (delete as necessary) section:

"We acknowledge funding from the South African Medical Research Council (https://www.samrc.ac.za/) (Reference number SHIPNCD 76756) [C. Cohen], The Wellcome Trust and the United Kingdom Foreign, Commonwealth and Development Office (https://wellcome.org/) (Grant no 221003/Z/20/Z) [C. Cohen] and United States Centers for Disease Control and Prevention (https://www.cdc.gov/) (Grant number 5 U01IP001048-05-00) [C. Cohen]. The funders had no role in study design, data collection and analysis, decision to publish, or preparation of the manuscript."

We note that you received funding from a commercial source: "Wellcome Trust"

Please provide an amended Competing Interests Statement that explicitly states this commercial funder, along with any other relevant declarations relating to employment, consultancy, patents, products in development, marketed products, etc.

**Reply:** Cheryl Cohen received funding from the Wellcome Trust. This has already been included in the Competing Interests Statement as highlighted in yellow: ”CC has received grant support from Sanofi Pasteur, South African Medical Research Council, The Wellcome Trust and the United Kingdom Foreign, Commonwealth and Development Office, PATH, US Centers for Disease Control and Prevention (CDC) and the Bill and Melinda Gates Foundation. NW and AvG have received grant support from Sanofi Pasteur, US Centers for Disease Control and Prevention (CDC) and the Bill and Melinda Gates Foundation.”

Within this Competing Interests Statement, please confirm that this does not alter your adherence to all PLOS ONE policies on sharing data and materials by including the following statement: "This does not alter our adherence to PLOS ONE policies on sharing data and materials.” (as detailed online in our guide for authors http://journals.plos.org/plosone/s/competing-interests). If there are restrictions on sharing of data and/or materials, please state these. Please note that we cannot proceed with consideration of your article until this information has been declared.

**Reply:** This sentence has been added to the Competing Interests Statement as follows: CC has received grant support from Sanofi Pasteur, South African Medical Research Council, The Wellcome Trust and the United Kingdom Foreign, Commonwealth and Development Office, PATH, US Centers for Disease Control and Prevention (CDC) and the Bill and Melinda Gates Foundation. NW and AvG have received grant support from Sanofi Pasteur, US Centers for Disease Control and Prevention (CDC) and the Bill and Melinda Gates Foundation. This does not alter our adherence to PLOS ONE policies on sharing data and materials.

Please include your amended Competing Interests Statement within your cover letter. We will change the online submission form on your behalf.

**Reply:** The amended Competing Interests Statement has been included in the cover letter, thank you.

3. Thank you for stating the following in the Competing Interests section:

"I have read the journal's policy and the authors of this manuscript have the following competing interests: CC has received grant support from Sanofi Pasteur, South African Medical Research Council, The Wellcome Trust and the United Kingdom Foreign, Commonwealth and Development Office, PATH, US Centers for Disease Control and Prevention (CDC) and the Bill and Melinda Gates Foundation. NW and AvG have received grant support from Sanofi Pasteur, US Centers for Disease Control and Prevention (CDC) and the Bill and Melinda Gates Foundation."

Please confirm that this does not alter your adherence to all PLOS ONE policies on sharing data and materials, by including the following statement: "This does not alter our adherence to PLOS ONE policies on sharing data and materials.” (as detailed online in our guide for authors http://journals.plos.org/plosone/s/competing-interests). If there are restrictions on sharing of data and/or materials, please state these. Please note that we cannot proceed with consideration of your article until this information has been declared.

**Reply:** This sentence has been added to the Competing Interests Statement as follows: CC has received grant support from Sanofi Pasteur, South African Medical Research Council, The Wellcome Trust and the United Kingdom Foreign, Commonwealth and Development Office, PATH, US Centers for Disease Control and Prevention (CDC) and the Bill and Melinda Gates Foundation. NW and AvG have received grant support from Sanofi Pasteur, US Centers for Disease Control and Prevention (CDC) and the Bill and Melinda Gates Foundation. This does not alter our adherence to PLOS ONE policies on sharing data and materials.

Please include your updated Competing Interests statement in your cover letter; we will change the online submission form on your behalf.

4. In your Data Availability statement, you have not specified where the minimal data set underlying the results described in your manuscript can be found. PLOS defines a study's minimal data set as the underlying data used to reach the conclusions drawn in the manuscript and any additional data required to replicate the reported study findings in their entirety. All PLOS journals require that the minimal data set be made fully available. For more information about our data policy, please see http://journals.plos.org/plosone/s/data-availability.

Upon re-submitting your revised manuscript, please upload your study’s minimal underlying data set as either Supporting Information files or to a stable, public repository and include the relevant URLs, DOIs, or accession numbers within your revised cover letter. For a list of acceptable repositories, please see http://journals.plos.org/plosone/s/data-availability#loc-recommended-repositories. Any potentially identifying patient information must be fully anonymized.

Important: If there are ethical or legal restrictions to sharing your data publicly, please explain these restrictions in detail. Please see our guidelines for more information on what we consider unacceptable restrictions to publicly sharing data: http://journals.plos.org/plosone/s/data-availability#loc-unacceptable-data-access-restrictions. Note that it is not acceptable for the authors to be the sole named individuals responsible for ensuring data access.

We will update your Data Availability statement to reflect the information you provide in your cover letter

**Reply:** The dataset used and analysed in this study has been uploaded to GitHub, and can be accessed using the following link: <https://github.com/crdm-nicd/huts_hus_2020_2021.git>. The data availability statement has also been updated.

5. We note that you have stated that you will provide repository information for your data at acceptance. Should your manuscript be accepted for publication, we will hold it until you provide the relevant accession numbers or DOIs necessary to access your data. If you wish to make changes to your Data Availability statement, please describe these changes in your cover letter and we will update your Data Availability statement to reflect the information you provide.

**Reply:** Thank you, we have made the data available, and have updated the data availability statement as follows: “The dataset used and analysed during the current study is available at <https://github.com/crdm-nicd/huts_hus_2020_2021.git>”

6. We note that Supplementary figures 1 and 2 in your submission contain [map, satellite] images which may be copyrighted. All PLOS content is published under the Creative Commons Attribution License (CC BY 4.0), which means that the manuscript, images, and Supporting Information files will be freely available online, and any third party is permitted to access, download, copy, distribute, and use these materials in any way, even commercially, with proper attribution. For these reasons, we cannot publish previously copyrighted maps or satellite images created using proprietary data, such as Google software (Google Maps, Street View, and Earth). For more information, see our copyright guidelines: http://journals.plos.org/plosone/s/licenses-and-copyright.

We require you to either (1) present written permission from the copyright holder to publish these figures specifically under the CC BY 4.0 license, or (2) remove the figures from your submission:

1. You may seek permission from the original copyright holder of Supplementary figures 1 and 2 to publish the content specifically under the CC BY 4.0 license.

We recommend that you contact the original copyright holder with the Content Permission Form (http://journals.plos.org/plosone/s/file?id=7c09/content-permission-form.pdf) and the following text:

“I request permission for the open-access journal PLOS ONE to publish XXX under the Creative Commons Attribution License (CCAL) CC BY 4.0 (http://creativecommons.org/licenses/by/4.0/). Please be aware that this license allows unrestricted use and distribution, even commercially, by third parties. Please reply and provide explicit written permission to publish XXX under a CC BY license and complete the attached form.”

Please upload the completed Content Permission Form or other proof of granted permissions as an "Other" file with your submission.

In the figure caption of the copyrighted figure, please include the following text: “Reprinted from [ref] under a CC BY license, with permission from [name of publisher], original copyright [original copyright year].”

2. If you are unable to obtain permission from the original copyright holder to publish these figures under the CC BY 4.0 license or if the copyright holder’s requirements are incompatible with the CC BY 4.0 license, please either i) remove the figure or ii) supply a replacement figure that complies with the CC BY 4.0 license. Please check copyright information on all replacement figures and update the figure caption with source information. If applicable, please specify in the figure caption text when a figure is similar but not identical to the original image and is therefore for illustrative purposes only.

The following resources for replacing copyrighted map figures may be helpful:

USGS National Map Viewer (public domain): http://viewer.nationalmap.gov/viewer/

The Gateway to Astronaut Photography of Earth (public domain): http://eol.jsc.nasa.gov/sseop/clickmap/

Maps at the CIA (public domain): https://www.cia.gov/library/publications/the-world-factbook/index.html and https://www.cia.gov/library/publications/cia-maps-publications/index.html

NASA Earth Observatory (public domain): http://earthobservatory.nasa.gov/

Landsat: http://landsat.visibleearth.nasa.gov/

USGS EROS (Earth Resources Observatory and Science (EROS) Center) (public domain): http://eros.usgs.gov/#

Natural Earth (public domain): <http://www.naturalearthdata.com/>

**Reply:** Supplementary figures 1 and 2 have been removed from the manuscript.

7. Please review your reference list to ensure that it is complete and correct. If you have cited papers that have been retracted, please include the rationale for doing so in the manuscript text, or remove these references and replace them with relevant current references. Any changes to the reference list should be mentioned in the rebuttal letter that accompanies your revised manuscript. If you need to cite a retracted article, indicate the article’s retracted status in the References list and also include a citation and full reference for the retraction notice.

**Reply:** The reference list has been reviewed and is complete and correct.

**Reviewers' comments:**

5. Review Comments to the Author

**Reviewer #1:** The research team has conducted a well-planned study of good quality to understand the healthcare utilization during covid-19 pandemic. The article is well written. I suggest reviewers to consider the following questions for further clarity.

1. Please indicate how the case definition for ILI and SRI were determined with references and include this information in the Methods section.

**Reply:** The references on which the case definitions for SRI and ILI were defined have been added to the methods section, page 8 as follows: “SRI and ILI case definitions were adapted from previous healthcare utilization surveys [2,5,10].”

2. The authors have mentioned BMI was calculated only for 5 years and above. Why was weight for age not considered for < 5-year-olds?

**Reply:** We were not able to calculate weight for age for children aged <5 years, as this calculation requires age in months, which was not available from the dataset. Only data in years was available and therefore BMI could only be calculated for individuals aged ≥5 years. This has been added to the methods, page 8: “BMI could not be calculated for children aged <5 years as age in month units was not available, and were classified as unknown.”

3. According to table 3, BMI was included as a dependent variable in the logistic regression analysis. How were unavailable BMI data from 1331 children <5 years compensated?

**Reply:** Individuals for whom BMI data was unavailable (including children aged <5 years) were classified as “unknown” in tables 2 and 3 (multivariable logistic regression analysis). This has been added to the methods, page 8: “BMI could not be calculated for children aged <5 years as age in month units was not available, and were classified as unknown.”

4. According to the analysis, 11.1% of those who died have not sought medical care and 37% have died at home. This could be a potential bias, underestimating the prevalence of people who did not seek healthcare during the specified time.

**Reply:** We agree with the reviewer and have added the following to the limitations paragraph, page 25: “Fourth, the number of individuals that did not seek healthcare for their illness is likely underestimated as individuals that had died during the study period were not included.”

5. Authors conclusion that community education is essential to promote timely presentation cannot be agreed with for two main reasons. Firstly, your study did not assess the effectiveness of community education on the timely presentation for healthcare, hence is not based on your findings. Secondly, the majority of your study sample did not utilize healthcare because they thought they were not sick enough. Do authors justify seeking early care for all respiratory symptoms or how does one draw the line when to seek care? How would this align with efficient healthcare resource utilization and burden on the health system? Therefore, revising the conclusion is highly recommended.

**Reply:** As suggested by the reviewer, we have removed this sentence from the conclusions paragraph on page 25.

**Reviewer #2:** This is an interesting and important paper which fills an information gap - the proportion of people with ILI and SRI that actually end up seeking healthcare during the COVID pandemic. It is useful evidence for interpreting sentinel site data. There are some minor clarifications to be made to further strengthen the paper.

Data available without restriction, but can only be given for researchers who meet criteria - is the first answer correct?

**Reply:** We have made the data publically available, and have updated the data availability statement as follows: “The dataset used and analysed during the current study is available at <https://github.com/crdm-nicd/huts_hus_2020_2021.git>”

Abstract

Should it be only ILI (0.7%), only SRI (0.8%)? (p3, line 45)

**Reply:** This sentence has been updated as suggested by the reviewer, abstract page 3.

Introduction

Is it possible to mention where the sentinel surveillance takes place (I assume “facilities” as mentioned in line 82?), to add more clarity to this paragraph? (p5, line 80)

**Reply:** This sentence has been updated as follows, page 5: “The National Institute for Communicable Diseases (NICD) has conducted syndromic surveillance for severe respiratory illness (SRI) and influenza-like illness (ILI) at sentinel hospitals and primary healthcare clinics in five provinces of South Africa since 2009”

Regarding the three sites surveyed (Mitchell’s Plain (Western Cape Province), Pietermaritzburg (KwaZulu-Natal Province), and Klerksdorp (North West Province)):

Can you provide some pertinent information about the three sites? e.g. are they similar to each other? Urban/rural? Area SES status, population density etc, so readers not familiar with the South African setting can get a better sense of each of your sites? You do explore the respondents’ characteristics by site in detail in the results, so a more general overview may be useful in the introduction. (p6, line 83)

**Reply:** We have added information on the three study sites in the methods section, page 6 as follows: “Mitchell’s Plain is a large township in the City of Cape Town, Western Cape Province, Pietermaritzburg is the capital and second largest city in KwaZulu-Natal Province, and Klerksdorp is the largest city in North West Province.”

Methods

Can you briefly describe why you choose those three communities (I understood that there are five provinces for sentinel surveillance?) (p6, line 95)

**Reply:** Sites were chosen based on having had a number of years of facility-based surveillance data available as well as having both primary healthcare clinics (ILI surveillance) and hospitals (SRI surveillance) sentinel sites. We have added the following to methods, page 6: “Study sites were selected based on having long-term surveillance data available from both primary healthcare clinic (ILI surveillance) and hospital (SRI surveillance) facilities.”

Do you have a source for these definitions of SRI and ILI? (p7/p8)

**Reply:** The references on which the case definitions for SRI and ILI were defined have been added to the methods section, page 8 as follows: “SRI and ILI case definitions were adapted from previous healthcare utilization surveys [2,5,10].”

You could add a comment that it is possible to have SRI without ILI if a person reported only pneumonia (is that the case?), so that the tables are easier to understand (p8, line 132).

**Reply:** The following has been added to methods, page 8: “If the participant reported >1 respiratory illness episode, information was collected on the most recent episode of respiratory illness.”

Healthcare seeking - what was the recall period? or was it specific to the seeking healthcare for the ILI/SRI illness? (p8, line 136)

**Reply:** Healthcare seeking was specific to the ILI/SRI illness episode reported by the participant. This has been clarified in methods, page 8: “Demographic information as well as information on underlying illnesses (including tuberculosis (current or previous), asthma, diabetes, chronic heart disease, chronic lung disease, hypertension and cancer), symptoms experienced and healthcare seeking for the reported ILI/SRI episode (clinic, hospital, traditional healer, religious leader, friend/relative, community health worker or pharmacy) were collected from participants.”

participants’ (p8, line 146)

**Reply:** This has not been changed as it is written as a plural, not as a possessive term in this context.

SES (p8, line 148) - could you include more detail on this in the supplement? Did you use PCA or some other method to create a score? Is the way you calculated SES validated in some way?

**Reply:** The reference used to determine SES has been added in methods, page 9: “Socioeconomic status (SES) was measured using a standardised set of questions described in previous research [13]”

Results

Is it “enrolled [participants] had a median age.. “ (p10, line 180)

**Reply:** Thank you for bringing this to our attention, we have added the word “participants”, page 14.

Healthcare seeking varied by site [please clarify for what]. (p11, line 206/7)

**Reply:** This sentence has been updated for clarification as follows, page 15: “The proportion of individuals that sought medical care varied by site (97/155 (62.6%) in Pietermaritzburg, 36/96 (37.5%) in Klerksdorp and 37/65 (56.9%) in Mitchell’s Plain, P<0.001.”

Died deaths —> died (p13, line 269)

**Reply:** Thank you, this has been corrected as suggested (page 22).

Discussion

Could you please provide some context on the 2012/3 surveys - were they done because of some respiratory illness that occurred during that time, hence higher numbers, or was it a based on routine surveillance? (p14, line 297-300)

**Reply:** The following sentence has been added to the discussion, page 23: “Previous healthcare utilization surveys have been conducted in sentinel site catchment populations in order to estimate the proportion of illness not captured by routine facility-based surveillance.”
